# Supplementary material for: Dual-Process Theory of Thought and Inhibitory Control: An ALE Meta-Analysis
Source: Brain Sci. 2024 Jan 20;14(1):101. doi: 10.3390/brainsci14010101 (PMC10813498; doi:10.3390/brainsci14010101)
Supplement: Supplementary file 1 [file brainsci-14-00101-s001.zip › Supplementary Table S1.pdf]

Table S1. Quality assessement of the included studies based guidelines for the standardized reporting of MRI studies (Poldrack, R. A. et al. Guidelines for reporting an fMRI study. Neuroimage 40, 409–414, 2008).

Study	Age reported	Sample gender reported	Sample handedness reported	Ethics approval reported	Image acquisition described	Image processing described	Statistical MRI analysis described	Software package specified	Multiple comparison correction
Goel et al. 2003	yes	yes	yes	yes	yes	yes	yes	yes	yes
Canessa et al. 2005	yes	yes	yes	yes	yes	yes	yes	yes	yes
Beierholm et al. 2011	no	no	no	yes	yes	yes	yes	yes	no
Liu et al. 2012	yes	yes	yes	yes	yes	yes	yes	yes	yes
Liang et al. 2014	yes	yes	yes	yes	yes	yes	yes	yes	yes
Liang et al. 2014	yes	yes	yes	yes	yes	yes	yes	yes	yes
Luo et al. 2014	yes	yes	yes	yes	yes	yes	yes	yes	yes
von Helversen et al. 2014	yes	yes	yes	yes	yes	yes	yes	yes	yes
Durning et al. 2015	yes	yes	yes	yes	yes	yes	yes	yes	yes
Megìas et al. 2015	yes	yes	no	yes	yes	yes	yes	yes	yes
Vartanian et al. 2018	yes	yes	yes	yes	yes	yes	yes	yes	yes
van den Berg et al. 2020	yes	yes	no	yes	yes	yes	yes	yes	yes
