# Supplementary material for: Dual-Process Theory of Thought and Inhibitory Control: An ALE Meta-Analysis
Source: Brain Sci. 2024 Jan 20;14(1):101. doi: 10.3390/brainsci14010101 (PMC10813498; doi:10.3390/brainsci14010101)
Supplement: Supplementary file 1 [file brainsci-14-00101-s001.zip › Supplementary Table S2.pdf]

Table S2. Studies contribution to the clusters.

**Contributors to cluster #1**
3 foci from Vartanian, 2018: The Reflective Mind: Examining Individual Differences in Susceptibility to Base Rate Neglect with fMRI. J Cogn Neurosci
1 foci from Megìas, 2015: Neural mechanisms underlying urgent and evaluative behaviors: An fMRI study on the interaction of automatic and controlled processes. Hum Brain Mapp
2 foci from Liang, 2014: Different neural systems contribute to semantic bias and conflict detection in the inclusion fallacy task. Front Hum Neurosci
1 foci from von Helversen, 2014a: Neural substrates of similarity and rule-based strategies in judgment. Front Hum Neurosci
2 foci from Beierholm, 2011: Separate encoding of model-based and model-free valuations in the human brain. Neuroimage
1 foci from Liang, 2014: Different strategies in solving series completion inductive reasoning problems: An fMRI and computational study. Int J Psychophysiol.
**Contributors to cluster #2**
1 foci from Vartanian, 2018: The Reflective Mind: Examining Individual Differences in Susceptibility to Base Rate Neglect with fMRI. J Cogn Neurosci
1 foci from Liang, 2014: Different neural systems contribute to semantic bias and conflict detection in the inclusion fallacy task. Front Hum Neurosci
2 foci from Beierholm, 2011: Separate encoding of model-based and model-free valuations in the human brain. Neuroimage
2 foci from Liang, 2014: Different strategies in solving series completion inductive reasoning problems: An fMRI and computational study. Int J Psychophysiol.
